# Identification of SOX2 Interacting Proteins in the Developing Mouse Lung With Potential Implications for Congenital Diaphragmatic Hernia

**DOI:** 10.3389/fped.2022.881287

**Published:** 2022-05-09

**Authors:** Kim A. A. Schilders, Gabriëla G. Edel, Evelien Eenjes, Bianca Oresta, Judith Birkhoff, Anne Boerema-de Munck, Marjon Buscop-van Kempen, Panagiotis Liakopoulos, Petros Kolovos, Jeroen A. A. Demmers, Raymond Poot, Rene M. H. Wijnen, Dick Tibboel, Robbert J. Rottier

**Affiliations:** ^1^Department of Pediatric Surgery, Erasmus Medical Center (MC)-Sophia Children’s Hospital, Rotterdam, Netherlands; ^2^Department of Molecular Biology and Genetics, Democritus University of Thrace, Alexandroupolis, Greece; ^3^Department of Biochemistry, Erasmus MC, Rotterdam, Netherlands; ^4^Department of Cell Biology, Erasmus MC, Rotterdam, Netherlands

**Keywords:** lung development, SOX2, congenital diaphragmatic hernia, CHD4, CUX1

## Abstract

Congenital diaphragmatic hernia is a structural birth defect of the diaphragm, with lung hypoplasia and persistent pulmonary hypertension. Aside from vascular defects, the lungs show a disturbed balance of differentiated airway epithelial cells. The Sry related HMG box protein SOX2 is an important transcription factor for proper differentiation of the lung epithelium. The transcriptional activity of SOX2 depends on interaction with other proteins and the identification of SOX2-associating factors may reveal important complexes involved in the disturbed differentiation in CDH. To identify SOX2-associating proteins, we purified SOX2 complexes from embryonic mouse lungs at 18.5 days of gestation. Mass spectrometry analysis of SOX2-associated proteins identified several potential candidates, among which were the Chromodomain Helicase DNA binding protein 4 (CHD4), Cut-Like Homeobox1 (CUX1), and the Forkhead box proteins FOXP2 and FOXP4. We analyzed the expression patterns of FOXP2, FOXP4, CHD4, and CUX1 in lung during development and showed co-localization with SOX2. Co-immunoprecipitations validated the interactions of these four transcription factors with SOX2, and large-scale chromatin immunoprecipitation (ChIP) data indicated that SOX2 and CHD4 bound to unique sites in the genome, but also co-occupied identical regions, suggesting that these complexes could be involved in co-regulation of genes involved in the respiratory system.

## Introduction

Congenital diaphragmatic hernia (CDH) is a structural birth defect of the diaphragm, and patients are characterized by lung hypoplasia and persistent pulmonary hypertension, which is associated with vascular abnormalities due to aberrant perivascular cells (1–4). In addition, CDH patients show an aberrant cellular composition of the airway epithelium, such as hyperplasia of the pulmonary neuroendocrine cell (PNEC) population and hypoplasia of club cell population (5–7). The conducting airway epithelium starts to differentiate before the alveolar epithelium, as shown in mouse development (8–13). The SRY-Related HMG-Box Gene 2 (SOX2) transcription factor is one of the earliest genes expressed in the proximal lung region and its expression is necessary and sufficient for the epithelial cells that mark the conducting airways (14–17). As the epithelial cells start to express SOX2, they become proximal airway and differentiate into basal cells, ciliated cells and club cells (15, 18). SOX2 directly regulates the transcription of the basal cell marker TRP63 and initiates the emergence of basal cells, via direct activation of the TRP63 promoter (15). Mice with altered SOX2 expression levels display aberrant epithelial differentiation, a phenotype that is frequently associated with pediatric lung diseases, such as in congenital pulmonary airway malformation (CPAM) and congenital diaphragmatic hernia (CDH) (19). Reduced levels of SOX2 during development leads to foregut defects resembling esophagus atresia with tracheoesophageal fistula and fewer basal cells (20). In contrast to reduced SOX2 levels, ectopic SOX2 expression in epithelial cells during development resulted in a decreased number of airways, enlarged airspaces and abnormal alveolar formation. Interestingly, the size of these cyst-like structures depended on timing and duration of ectopic SOX2 expression (15). Aside from these structural abnormalities, cellular changes of the epithelium were notable with an increase in basal cells and neuroendocrine cells (14).

The transcriptional activity of SOX2 depends on its interaction with other proteins, leading to “complex-specific” DNA binding and subsequent transcriptional regulation. Some of the earliest identified partners are OCT3/4, which plays a role in stem cell regulation, and PAX6, which has a role in lens development (21–23). Previously, we described two novel partners, XPO4, which is involved in nucleo-cytoplasmic trafficking (24), and CHD7, a chromatin remodeling ATPase associated with CHARGE syndrome (25). However, for lung development it is still unknown which specific SOX2 partners are involved in the differentiation of proximal epithelium, and if these partners may be important in aberrant differentiation of the epithelium in CDH.

Therefore, we used a specific mouse model expressing a biotinylatable SOX2, the bioSOX2 mouse, to isolate SOX2 complexes *in vivo* during airway epithelium differentiation (26). We identified several interacting proteins and showed that CHD4, FOXP2, FOXP4, and CUX1 act as SOX2 interacting partners during lung development. We confirmed co-localization of these proteins with SOX2 in the trachea and upper airways, confirmed their interaction with SOX2 and, for CHD4, we showed potential co-regulation of target genes *in vivo*.

## Materials and Methods

### bioSOX2/birA Mice

BioSOX2 and birA mice were previously generated and maintained on C57/Bl6 background under standard conditions and experiments were performed following guidelines of the ethics committee of the Erasmus Medical Center (26, 27).

### Immunohistochemistry

Lungs from wild type mouse embryos from E11 till E18 were dissected and fixed in 4% PFA overnight at 4°C before processing for paraffin embedding. Sections of 5 μm were dewaxed and rehydrated, followed by antigen retrieval with microwave treatment in 10 mM Tris-HCl, 1 mM EDTA pH9.0 (TE). After quenching endogenous peroxidase with 1.5% H_2_O_2_, samples were blocked with 1% BSA, 0.05% Tween20 in PBS for 10 min at room temperature and then incubated with antibody against CHD4 (Abcam; ab72418), FOXP2 (Abcam; ab16046), FOXP4 (Santa Cruz; sc-292474), or CUX1 (Abcam, Ab54583) diluted in blocking buffer at 4°C overnight. After washing in PBS with 0.05% Tween20, slides were incubated in blocking buffer with biotin conjugated secondary antibody for 30 min at room temperature. Then samples were incubated with VECTASTAIN^®^ ABC Reagent (Vector) for 30 min, followed by incubation with diaminobenzidine (Fluka). Slides were counterstained with hematoxylin and mounted with pertex. Sections were analyzed using Olympus BX41 microscope.

### Immunofluorescent Staining

Lungs from wild type mouse embryos from E11 till E18 were dissected and fixed in 4% PFA overnight at 4°C before processing for paraffin embedding. Sections of 5 μm were dewaxed and rehydrated, followed by antigen retrieval with microwave treatment in TE. Samples were blocked and incubated with antibodies against CHD4 (ab72418) and SOX2 (GTI5098), FOXP2 (ab16046) and SOX2, and CUX1 (Abcam, Ab54583) and SOX2 diluted in blocking buffer at 4°C overnight. After washing in PBS with 0.05% Triton X-100, slides were incubated in blocking buffer with fluorophore conjugated secondary antibody (Alexa Fluor-488, Alexa Fluor-594; Jackson Immuno Research) for 1 h at room temperature. Slides were mounted in Vectashield Mounting Medium with Dapi (Vector laboratories, Burlingame, CA, United States). Digital images were captured using a ZEISS imager Z1 AX10 microscope.

### Cell Culture, Co-transfections, and Co-immunoprecipitations

HEK293 (HEK) cells were cultured in DMEM (Lonza, Verviers, Belgium) with 5% fetal calf serum and 1% penicillin-streptomycin under standard culture conditions. NCCIT cells were cultured in DMEM (Lonza, Verviers, Belgium) with 10% fetal calf serum and 1% penicillin-streptomycin under standard culture conditions.

HEK cells were transfected with different combinations of pcDNA3 based plasmids expressing FLAG-tagged CHD4, FLAG-HA-tagged WDR5, myc-tagged SOX2, myc-tagged FOXP2, myc-tagged FOXP4, myc-tagged TCF3, and FLAG-tagged SOX2, using X-tremeGENE HP DNA Transfection Reagent (Roche, Basel, Switzerland). Similar co-transfections and co-immunoprecipitations were performed using pcDNA3-myc-HA-tagged CUX1 and different SOX2 constructs that were described previously (24, 28–34). X-tremeGENE HP DNA Transfection Reagent (Roche, Basel, Switzerland) was used for the transfection according to the manufacturer’s manual. Cells were harvested 24 h after transfection. Total cell extracts were prepared in 300 μl cell lysis buffer (20 mM Tris pH8, 137 mM NaCl, 10 mM EDTA, 1% NP40, 10% glycerol) with Complete protease inhibitor (Roche, Basel, Switzerland). 50 μl extract was incubated for 2 h at 4°C in 250 μl cell lysis buffer with antibodies against myc (Roche; 1668149) and FLAG (Sigma-Aldrich; F1804), followed by 1 h incubation with protein G beads (Sigma-Aldrich, St. Louis, MO, United States). After washing with cell lysis buffer, the beads were resuspended in 20 μl sample buffer and heated for 10 min at 95°C.

### Western Blotting

Samples were separated on a SDS-PAGE gel and transferred to a PVDF membrane by wet blotting for 2 h at 100 V and 400 mA. Membranes were blocked in TBS with 0.05% Tween-20 and 5% BSA. Membranes were labeled with antibodies against myc (Abcam; ab9106) and FLAG (Sigma-Aldrich; F7425) for 2 h, followed by an 1 h incubation with secondary HRP-labeled antibody. Membranes were developed with ECL incubation (Thermo Fisher Scientific INC., Waltham, MA, United States) on an Alliance Imager (Uvitec, Cambridge, United Kingdom).

### Glutathione S-Transferase-Fusion Tags

Generation of glutathione S-transferase (GST) fusion-tags and bacterial lysates is previously described (24, 35). HEK cells were grown ∼85% confluent and nuclear extracts were prepared. 10 μg of the bacterial lysate was incubated with 25 μl glutathione sepharose UB beads (GE Healthcare)/1% fish skin gelatin for 2 h rotating at 4°C. Beads are washed 4x with bacteria lysis buffer (20 mM Hepes-NaOH pH7.6, 150 mM KCl, 10% glycerol, 0.5 mM EDTA, 10% triton; 0.5 mM DTT + 1x CEF prior to use), 2x with wash buffer (20 mM Hepes pH7.6, 20% glycerol, 100 mM KCl, 1.5 mM MgCl_2_, 0.2 mM EDTA, 0.02% NP-40; 1x CEF prior to use) and are then incubated for 2 h with nuclear cell extracts rotating at 4°C. After washing 4 x with wash buffer, 40 μl 2x sample buffer is added and beads are heated for 10 min at 95°C. Western blotting was used to detect SOX2 binding partners.

### Chromatin Immunoprecipitation and Data Analysis

∼120 × 10^6^ NCCIT cells were double cross-linked with 2 mM disuccinimidyl glutate (Thermo Fisher Scientific) and 1% formaldehyde, or single cross-linked with 1% formaldehyde (36). Cells were lysed in ChIP cell lysis buffer (10 mM Tris pH 8.0, 10 mM NaCl, 0.2% NP-40, 1x CEF), followed by lysis of the nuclei in ChIP nuclei lysis buffer (50 mM Tris pH 8.0, 10 mM EDTA, 1% SDS, 1x CEF). Samples were sheared with a multiprobe bioruptor (double cross-linked: 75 min, 30 s high, 30 s off; single cross-linked: 30 min, 30 s high, 30 s off). Samples were diluted 10x with ChIP dilution buffer (16.7 mM Tris-HCl pH 8.1, 167 mM NaCl, 1.2 mM EDTA, 1.1% TritonX-100, 0.01% SDS), precleared and incubated O/N with antibodies against SOX2 (#2748 cell signaling) and CHD4 (Abcam, ab72418), and goat IgG (Santa Cruz) and rabbit IgG (Santa Cruz) as negative control. Samples were incubated with Protein A/G agarose beads 1 h rotating at 4°C and then washed with Low Salt Immune Complex Buffer (20 mM Tris-HCl pH8.0, 150 mM NaCl, 2 mM EDTA, 0.1% TritonX-100, 0.1% SDS), High Salt Immune Complex Buffer (20 mM Tris-HCl pH8.0, 500 mM NaCl, 2 mM EDTA, 0.1% TritonX-100, 0.1% SDS), LiCl Immune Complex Buffer (10 mM Tris-HCl pH8.0, 1 mM EDTA, 0.25 M LiCl, 1% NP-40, 1% deoxycholate), and TE (10 mM Tris-HCl pH8.0, 1 mM EDTA pH 8.0). Bound chromatin was eluted with freshly prepared elution buffer (1% SDS, 0.1 M NaHCO_3_), de-cross-linked and purified DNA was sequenced. For ChIP-PCR, the purified DNA was analyzed using gene-specific primer sets in a qPCR and the ΔC(t) method was used to determine the enrichment of the SOX2 and CHD4 precipitated DNA relative to the IgG control samples. ChIP-seq analysis was mainly performed as previously described (37). Peaks were called with the following criteria: peak ≥ 20, FDR ≤ 0.001, fold change over control ≥ 2. The iRanges package and the “findOverlaps” function from the GenomicRanges package (38) was used to identify the target genes and the overlap between the binding regions.

## Results

### Selected SOX2 Interacting Proteins Co-localize With SOX2 in the Developing Lung

The transcription factor SOX2 is expressed early in the developing lung and is one of the first molecular signs of epithelial cells that are committed to the proximal airway fate. To identify molecular switches that hallmark the differentiation of the proximal cell toward the different cell lineages eventually covering the upper airways, such as ciliated cells, club cells and basal cells, we generated a mouse line expressing a biotinylatable tagged SOX2 (26). An additional mouse strain ubiquitously expressing the bacterial biotin birA ligase was used to biotinylate the bioSOX2 protein *in vivo* (27). The biotin-tagged SOX2 is subsequently purified with high affinity using streptavidin, which has a much higher affinity than regular antibodies have for antigens (39). Using this mouse strain, we performed a large-scale screen to identify *in vivo* SOX2 interacting proteins using embryonic day 18.5 (E18.5) mouse lungs. Biotinylated SOX2 and associated proteins were precipitated with streptavidin magnetic beads and subsequently identified by mass spectrometry. A list of possible candidate partners of SOX2 was created based on the mascot score and peptide enrichment. From this list, a select number of proteins that could be of importance for lung epithelial differentiation were selected for further analysis (Table 1).

**TABLE 1:** Purified SOX2 associating proteins from E18.5 lungs.

	Mascot	Peptides
CHD4	1343 (939)	26 (21)
CUX1	476 (N.A.)	8 (0)
FOXP2	61 (N.A.)	2 (0)
FOXP4	86 (N.A.)	2 (0)

*Mascot scores and number of peptides for four SOX2 associating proteins are provided, with the control numbers between brackets (mass spec of control mouse lungs).*

**FIGURE 1:**
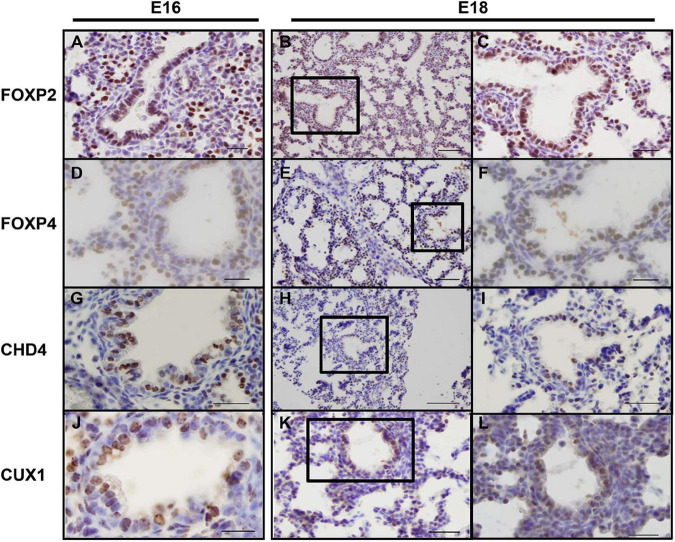
Endogenous CHD4, FOXP2/4, and CUX1 expression at E16 and E18 in mice. **(A–C)** At E16 **(A)** and E18 **(B,C)** FOXP2 is exclusively expressed in the distal airway epithelium. **(D–F)** At E16 **(D)**, FOXP4 expression becomes restricted to the epithelial cells and this pattern remains at E18 **(E,F)**. **(G–I)** CHD4 is primarily expressed in the conducting airways of the lung at E16 **(G)** and E18 **(H,I)**. **(J–L)** At E16 and E18, CUX1 is expressed in epithelial cells of the conducting airways and surrounding alveolar regions. Sections are frontal and scale bars are 10 μm **(B,E,H,K)** and 5 μm (others). Black boxes indicate magnified areas.

**FIGURE 2:**
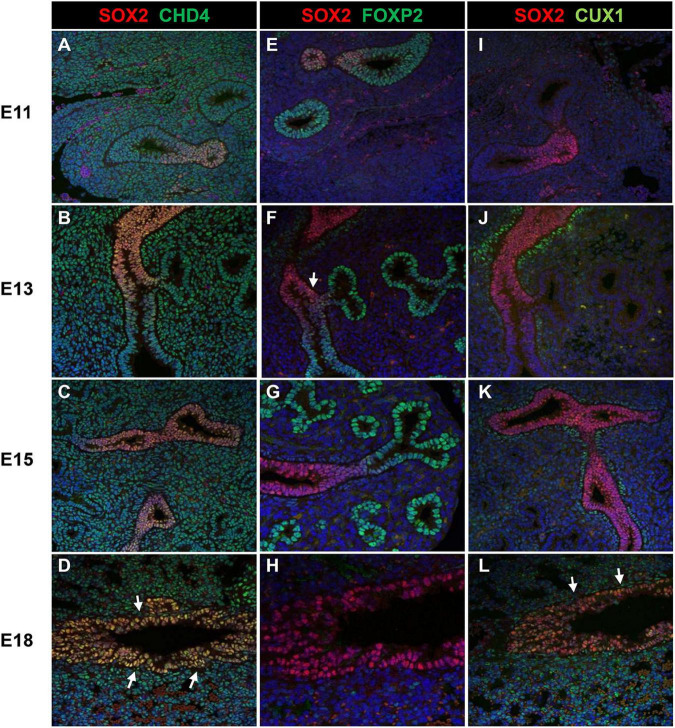
Co-localization of SOX2 with CHD4, FOXP2, or CUX1. **(A–D)** Co-localization of SOX2 and CHD4 in the proximal airways at different gestational ages (E11–E18). CHD4 and SOX2 are co-localized in the nuclei of the epithelial cells that line the proximal airways at E18 (red = SOX2, green = CHD4, blue = DAPI). **(E–H)** Co-localization of SOX2 and FOXP2 from E11 until E18 in the developing mouse lung. During E11–E13, some epithelial cells localized in the branching site between the proximal and distal region show co-localization of SOX2 and FOXP2 (red = SOX2, green = FOXP2, blue = DAPI). White arrows indicate co-localization. **(I**–**L)** SOX2 and CUX1 co-localize in the lung epithelium at E16 and E18. CUX1 and SOX2 co-localize in the nuclei of epithelial cells that line the proximal airways during E18. The arrows indicate the epithelial cells that express both SOX2 (red) and CUX1 (green).

As the screen for potential SOX2 interactors was based on nuclear protein extracts of total embryonic lungs, we first analyzed the spatial and temporal expression pattern of selected proteins during lung development in mice using immunohistochemistry. The Forkhead box protein FOXP2 expression was restricted to the distal lung epithelium during the late pseudoglandular, canalicular, and saccular phases of lung development (Figures 1A–C) and FOXP4 expression was first detected at E14 in the epithelium of the developing airways and the surrounding mesenchyme, but the expression became restricted to the proximal and distal airway epithelium during the canalicular phase (40). A similar expression pattern persisted in the saccular phase and postnatally (Figures 1D–F). FOXP2 and SOX2 were co-expressed in cells localized at the branching region between the proximal and distal region during the embryonic and early pseudoglandular phases of lung development (Figures 2E,F; arrow). During later stages of lung development, FOXP2 is restricted to the distal epithelial cells, while SOX2 is restricted to the proximal epithelial cells (Figures 2G,H). CHD4, one of the catalytic subunits of the nucleosome remodeling and deacetylase (NuRD) complex, was detectable in the embryonic lung until E16, but became significantly expressed in the epithelial cells of the proximal airways from the canalicular stage onward (Figures 1G–I). In the adult lung, CHD4 remained expressed in the epithelial cells of the large airways albeit at lower level (data not shown). CHD4 colocalized at E18 with SOX2 in the epithelial cells lining the proximal airways, thus, at the final stages of embryonic lung development, the spatial and temporal expression patterns of SOX2 and CHD4 overlap (Figures 2A–D; arrows). The Cut-Like Homeobox1 (CUX1) was initially expressed in the mesenchyme at the earliest stages of lung development until E16, but during the canalicular and saccular phases of the lung significant expression was detected in the epithelial cells of the airways (Figures 1J–L). In the adult mouse lung, CUX1 remained exclusively expressed in the epithelial cells of the large airways. CUX1 expression colocalized with SOX2 in epithelial cells of the large conducting airways at the canalicular stage (E16.5) and saccular stage (E18.5, Figures 2I–L). In conclusion, the spatial and temporal expression of these potential SOX2 interacting proteins correspond to the pattern of SOX2 expression.

### FOXP2, FOXP4, CUX1, and CHD4 Physically Interact With SOX2

**FIGURE 3:**
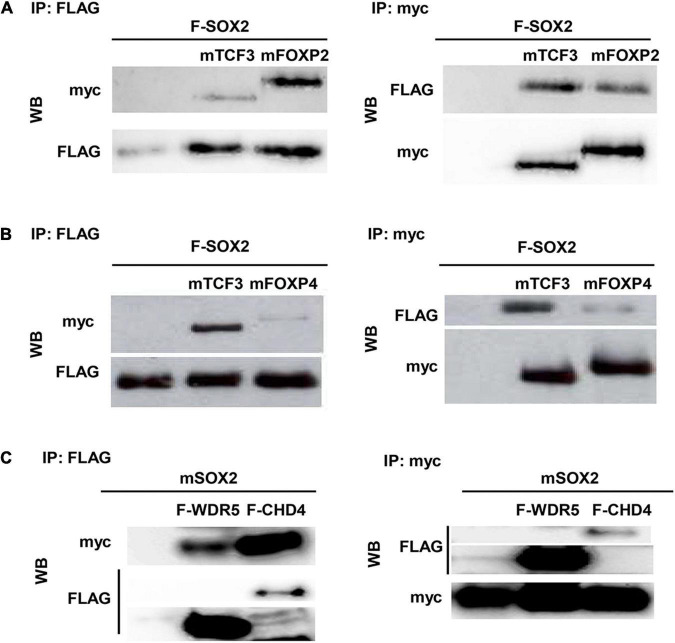
CHD4, FOXP2, and FOXP4 interact with SOX2. Interaction between FOXP2 **(A)**, FOXP4 **(B)**, and CHD4 **(C)** with SOX2 was confirmed in co-immunoprecipitations after transient transfection of expression constructs in HEK cells. FLAG-SOX2 and myc-FOXP2 **(A)**, FLAG-SOX2 and myc-FOXP4 **(B)** or myc-SOX2 and FLAG-CHD4 **(C)** were transfected and immunoprecipitations were done with antibodies against the different protein tags (myc or FLAG). Subsequently, Western blots were probed with antibodies against either FLAG of myc.

Since the spatial and temporal expression of SOX2 and its putative partners overlap, the direct interaction between the proteins was validated. Therefore, transient transfections were performed using constructs to express epitope-tagged proteins in different combinations. Immunoprecipitation of a FLAG-tagged SOX2 and either a myc-tagged isoform of FOXP2 or FOXP4 showed that SOX2 interacts with FOXP2 and FOXP4 (Figures 3A,B). Comparable immunoprecipitations were performed to validate the interaction between SOX2 and CHD4 using a FLAG-tagged CHD4 and a myc-tagged SOX2 protein, indicating that SOX2 and CHD4 also interact (Figure 3C). The interactions between SOX2 and TCF3 or WDR5 were used as positive controls, as we previously showed that these proteins interact with SOX2 (26).

**FIGURE 4:**
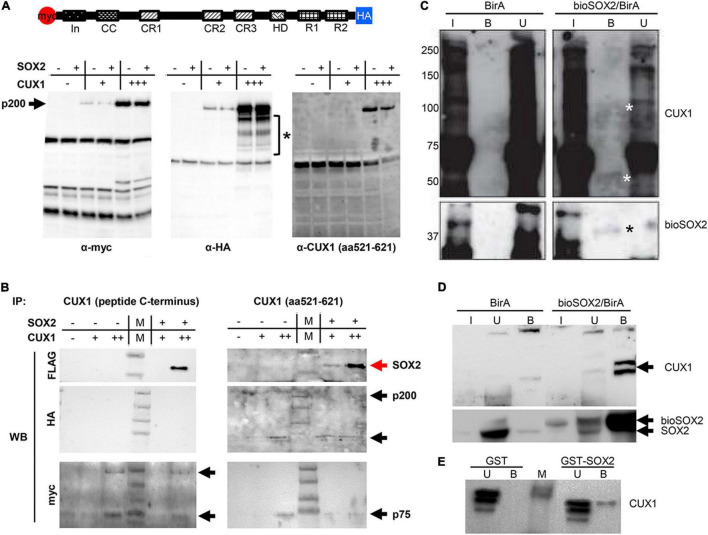
CUX1 has different isoforms which interact with SOX2. **(A)** Schematic representation of the CUX1 protein, N-terminally tagged with a myc peptide, and C-terminally with a HA peptide. The tagged CUX1 was co-transfected with a FLAG-tagged SOX2 construct, and total input was analyzed with antibodies against the myc-tag (α-myc), the HA-tag (α-HA), or a CUX1 raised against a peptide mapping at the C-terminus [α-CUX1 (aa521-621)] asteriks indicates CUX1 isoforms. **(B)** HEK cells were transfected with myc-CUX1-HA alone or together with FLAG-SOX2, and protein extracts were subsequently precipitated with one of two CUX1 antibodies (CUX1 peptide C-terminus or CUX1 aa521-621). Western blots were subsequently labeled with antibodies against the FLAG peptide to identify co-precipitating SOX2 protein, or with antibodies against the myc or HA tag to detect the CUX1 protein isoforms. Red arrows indicate the precipitated SOX2, black arrows indicate the precipitated CUX1 isoforms. **(C)** Protein extracts of tracheas isolated from birA mice (BirA) or bioSOX2/birA mice were incubated with streptavidin beads to isolate biotinylated SOX2 proteins. Total input (I), bound (B), and unbound (U) fractions were immunoblotted with antibodies against CUX1 (top, white asterisks indicate specific bands in the bound fraction) or against SOX2 (bottom, black asterisks). **(D)** Identical streptavidin precipitation as in panel **(C)**, using brain protein extracts. **(E)** Bacterial produced glutathione S-transferase (GST) or GST-fused to full length SOX2 (GST-SOX2) were incubated with nuclear protein extracts of HEK cells. CUX1 specifically precipitated with the GST-SOX2 fusion protein. U = eluate, B = precipitated fraction.

CUX1 is a large, 200 kDa protein, which is proteolytically processed both at the N- and C–terminus into several isoforms (41). We used a full length CUX1 expression construct containing an N-terminal myc-tag and a C-terminal HA-tag and analyzed which isoforms would be precipitated with the antibodies against the different tags (41). Immunoprecipitation of extracts from transient transfected HEK cells using antibodies against the myc- or HA tag showed that the full length CUX1 protein is proteolytically processed at both termini, as the myc and HA antibody detected different CUX1 isoforms (Figures 4A,B). The myc antibody detected isoforms containing the N-terminus, such as the full-length CUX1 protein (p200) and a p55 isoform, whereas the HA antibody detected different isoforms with the C-terminus intact (Figure 4A, asterisk). Thus, the full length p200 isoform generates multiple isoforms, presumptively corresponding to the p150, p110, p90, p80, and p75 isoforms as previously shown (Figure 4A, bracket; 41). In addition, a commercial CUX1 specific antibody raised against amino acids 521-621, a region between cut repeat 1 and 2 (CR1 and CR2), primarily recognized the p200 isoform (Figure 4A, right panel). To avoid missing specific interactions between CUX1 and SOX2, we used this antibody and another commercial antibody raised against the C-terminus of CUX1 to perform immunoprecipitations. Both antibodies precipitated the transiently transfected CUX1 protein, and efficiently co-precipitated the SOX2 protein, indicating that CUX1 and SOX2 interact (Figure 4B). Given the complex pattern of processed CUX1 isoforms, we decided to perform a streptavidin precipitation of embryonic trachea and brain protein extracts from birA and bioSOX2/birA mice to verify the interaction between SOX2 and CUX1. Comparing the birA with the bioSOX2/birA, it is clear that the biotinylated SOX2 is specifically precipitated, although the trachea showed lower amounts reflecting the fact that the trachea consists of several cell types, of which some express SOX2 (Figures 4C,D). Concomitantly, CUX1 is unambiguously co-precipitated, albeit more abundantly in the brain than in the trachea (Figures 4C,D, arrows). Finally, a bacterial produced GST-SOX2 fusion protein was used to investigate the nature of the interaction between SOX2 and CUX1 (24, 35). Nuclear extracts of HEK cells were incubated with GST-only or the GST-SOX2 fusion protein, resulting in a SOX2 specific precipitation of the p75 CUX1 isoform (Figure 4E). Collectively, these data show that CUX1 directly interacts with SOX2 in the developing airway epithelium.

**FIGURE 5:**
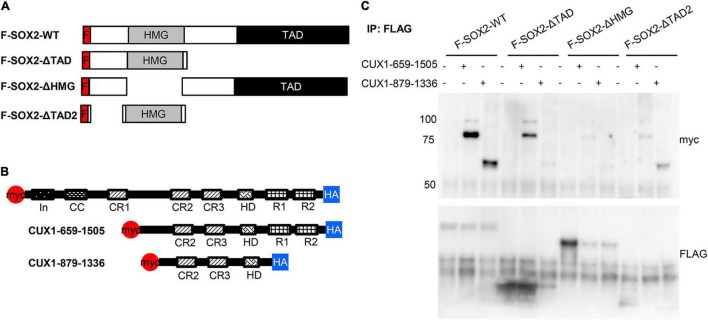
The N-terminal domain and DNA-binding domain of SOX2 are necessary for interaction with CUX1. **(A)** Co-transfections of mutant SOX2 constructs with CUX1 isoforms to identify the regions important for the SOX2-CUX1 interaction. Schematic representation of FLAG-tagged SOX2 constructs: full length SOX2 (F-SOX2-WT), SOX2 lacking the transactivation domain (F-SOX2-ΔTAD), SOX2 lacking the HMG DNA binding domain (F-SOX2-ΔHMG), and SOX2 lacking N-terminal amino acids 8-40 and the transactivation domain (F-SOX2-ΔTAD2) (33). These SOX2 variants were co-transfected in HEK cells with two CUX1 isoforms, CUX1 659-1505 and CUX1 879-1336 **(B)**, and protein extracts were used to perform immunoprecipitations followed by Western blot analysis. **(C)** Immunoprecipitation with a FLAG antibody shows specific precipitation of the CUX1 isoforms with F-SOX2-WT, and with the SOX2 isoform which still has the HMG DNA binding domain.

Previously, we showed that the HMG domain of SOX2 is important for the interaction with another interacting protein, XPO4 (24). Therefore, we used three SOX2 deletion proteins to analyze potential domains of interaction (Figure 5A). As we already showed the interaction of SOX2 with the full length CUX1, we also used two constructs expressing CUX1 truncated proteins based on their natural processing (33; Figure 5B). The full length SOX2 interacts with both CUX1 isoforms, but deletion of the transactivating domain of SOX2 results in reduced association of the short CUX1 isoform. This is even more pronounced when the amino acids 8–40 are removed (F-SOX2-ΔTAD2). Removal of the HMG domain completely abolishes the interaction with the CUX1 isoforms (Figure 5C). Collectively, these data show that the N-terminal and DNA-binding HMG domain of SOX are important for the interaction with CUX1. Moreover, the CUX1 domains that interact with SOX2 are within the short isoform of CUX1 containing the CR2, CR3, and HD domains.

### SOX2 and CHD4 Have Unique and Common Genomic Targets

**FIGURE 6:**
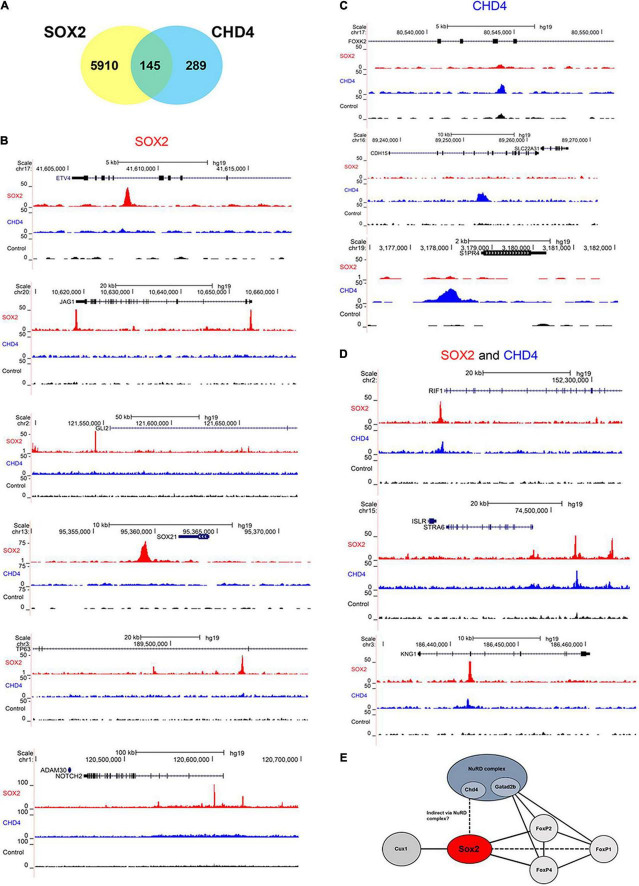
SOX2 and CHD4 bind same genomic regions. **(A)** Venn diagram showing the number of overlapping called ChIP peaks of SOX2 and CHD4. **(B)** UCSC browser plots showing SOX2 specific ChIP peaks (red) for ETV4, JAG1, GLI2, SOX21, TP63 (ΔNTP63 isoform promoter), and NOTCH2. **(C)** UCSC browser plots showing CHD4 specific ChIP peaks (blue) for FOXK2, CDH15, and S1PR4 (top to bottom). **(D)** UCSC browser plots showing genomic sites that are bound by SOX2 (red) and CHD4 (blue) for RIF1, STRA6, and KNG1. **(E)** Model to show the relations between several of the identified SOX2 interacting proteins.

Lastly, we performed a chromatin immunoprecipitation (ChIP) followed by sequencing, to identify if the SOX2 interacting protein CHD4 would also bind to DNA in close vicinity of SOX2. Therefore, the human pluripotent embryonal teratocarcinoma NCCIT cell line, which endogenously expresses SOX2 and CHD4, was used (Figure 6A). In total, over 6000 SOX2 specific binding sites were identified and just over 400 sites for CHD4. Gene ontology analysis showed that some of the identified binding sites that were in close proximity to transcriptional start sites in the SOX2 ChIP are associated with lung development, such as ETV4, JAG1, GLI2, SOX21, TP63 (ΔNTP63 isoform promoter), and NOTCH2 (Figure 6B, top to bottom). Other interesting lung-associated genes are HHIP, PDPN, BMPR1A, and EPAS1. For CHD4 we identified fewer binding sites, of which FOXK2, CDH15, and S1PR4 are shown (Figure 6C, top to bottom). Of the specific binding sites, 145 binding sites were shared by SOX2 and CHD4, of which RIF1, STRA6, and KNG1 are interesting examples (Figure 6D, top to bottom). Overall, we highlight here several examples of genes which may be regulated by SOX2 alone or in combination with CHD4.

## Discussion

SOX2 is an important transcription factor during lung development and an early marker of airway epithelium (14, 42, 43). Changes in the level of SOX2 during lung development leads to aberrant epithelial differentiation, as frequently associated with pediatric lung diseases, such as in congenital pulmonary airway malformation (CPAM) and congenital diaphragmatic hernia (CDH). CDH patients show an altered cellular composition of the airway epithelium, such as hyperplasia of the pulmonary neuroendocrine cell (PNEC) population and hypoplasia of the club cell population (5–7). The transcriptional activity of SOX2 largely depends on its interaction with other proteins and the identification of SOX2-associating proteins may be important to understand this aberrant epithelial differentiation in pediatric lung diseases. Using a mouse strain with a biotinylatable-tag inserted in the endogenous SOX2 locus to purify SOX2-containing complexes in the lung *in vivo*, we identified FOXP2, FOXP4, CHD4, and CUX1 as SOX2-associating proteins (Figure 6E).

Three candidate SOX2 partner proteins, FOXP1, FOXP2, and FOXP4, are involved in various biological processes, including lung development. In this study, interaction of SOX2 with both FOXP2 and FOXP4 was validated. During lung development these FOXP factors have an overlapping expression pattern and specific dimer combinations of FOXP1, FOXP2, and FOXP4 regulate subsets of target genes, including genes of the NOTCH and WNT signaling pathway (44). Interplay between FOXP1 and FOXP2 is necessary for proper lung airway morphogenesis and development of the esophagus (45). A conditional knock-out mouse of both factors results in a loss of secretory cells, indicating that FOXP1/4 is involved in secretory cell fate during development and repair (46). Since these cells are derived from SOX2^+^ progenitor cells, a potential interplay between SOX2 and FOXP1/4 could be involved in this process. Although SOX2 is restricted to the proximal airways and FOXP2 to the distal airways during later stages of development, co-localization was observed during the embryonic and early pseudoglandular phase. These double positive cells were localized in the branching site between the proximal and distal region, where also broncho-alveolar stem cells are located, suggesting that the FOXP2-SOX2 complex could be involved in early branching in lung development (47).

Another candidate binding partner identified is the Cut-Like Homeobox1 (CUX1), which we previously also identified as a potential SOX2 partner in neural stem cells (25). CUX1 is expressed in many cell types and organs, including the lung, and is involved in diverse processes, such as cell migration, cell adhesion and motility and it is involved in brain and liver development and cancer. Several CUX1 mutant mice have been generated, and one of these models has shown a role for CUX1 in lung development. Cutl1^Z/Z^ mice, which express an inactive CUX1 protein, die shortly after birth of respiratory distress and showed an aberrant lung epithelium, resulting in a thick, non-functional epithelium. The authors suggested that CUX1 is important for the differentiation of the cuboidal epithelial precursor cells into functional alveolar type I and II cells (48).

SOX2 and CUX1 physically interact and this interaction is dependent on the N-terminal and DNA-binding domain of SOX2 (Figure 5C; 33). The same domain is also involved in the interaction with other proteins, such as HDAC2 and XPO4 (24, 33). Previously, it is shown that SOX2 and CUX1 bind to several common target genes, including CHN1 (involved in Duane Retraction Syndrome), HOMER2 (linked to Congenital diaphragmatic hernia), HIF1AN and KCCN2, WDR37, FOXP4, ARL6 (involved in Bardet-Biedl syndrome) (49, 50), and several WNT genes in mouse mammary tumors (51). Altogether, this suggest that CUX1 plays a role in the developing epithelium by interaction with SOX2, regulating downstream target genes for epithelial differentiation, and interestingly, one of which is linked to CDH.

CHD4 is one of the catalytic subunits of the nucleosome remodeling and deacetylase (NuRD) complex, which makes DNA accessible to proteins and protein complexes to mediate several processes including transcription and repair (52). Other components of this complex are HDAC1/2, which are histone deacetylases, the non-enzymatic proteins MBD2/3, the retinoblastoma-binding proteins MTA1/2/3 and GATAD2A/B (52). Several studies have shown potential interaction between SOX2 and MTA1/2/3, MBD2, GATAD2A/B, and HDAC1/2 in different cell lines, including embryonic stem cells, neural stem cells, and embryonal carcinoma cells (25, 33). The NuRD complex is also involved in epithelial injury response in the lung by interacting with the FOXP1/2/4 family, suggesting that this complex is involved in lung injury repair and regeneration (53). Premature born babies that need oxygen supply after birth are mechanically ventilated and thereby exposed to high concentrations of oxygen, causing lung injury (54). The same holds true for the hypoplastic lungs of patients with CDH who develop chronic lung disease in thirty percent of the survivors. Based on the findings of Chokas and colleagues, the NuRD complex and its core component CHD4 could be involved in repair of these injured lungs, making it worthwhile to further investigate the role of CHD4 as a potential partner of SOX2 during lung development.

We also showed that CHD4 and SOX2 bind to the same genomic regions as SOX2, suggesting that the SOX2-CHD4 complex regulates genes that may be involved in the respiratory system.

In conclusion, we identified SOX2 interacting proteins during lung development, which may be important for the differentiation of the airway epithelium. For some, there are indications that these proteins may be associated with pediatric lung diseases, such as CDH. Previously, we showed that CHD7, which is linked to CHARGE syndrome, directly interacts with SOX2, which in turn is linked to AEG syndrome (25). Interestingly, CHARGE and AEG syndromes are frequently confused because of their overlapping clinical presentation, and so, the SOX2-CHD7 interaction may explain this confusion. This, in turn, supports the potential importance of our current findings in relation to understanding the involvement of SOX2 associating proteins in the occurrence of pulmonary epithelium defects in CDH.

## Data Availability Statement

The original contributions presented in the study are publicly available. This data can be found here: BioProject ID PRJNA816351 (https://www.ncbi.nlm.nih.gov/sra/PRJNA816351).

## Ethics Statement

The animal study was reviewed and approved by Ethics Committee of the Erasmus Medical Center.

## Author Contributions

KS, GE, EE, BO, JB, AB-dM, and MB-vK: experimental design, data acquisition, and analysis. PK, PL, JD, RP, and RR: proteomics and genomics analysis. RR: overall project design and financial acquisitions. RW, DT, and RR: management. All authors: writing, correcting, and approval of final manuscript.

## Conflict of Interest

The authors declare that the research was conducted in the absence of any commercial or financial relationships that could be construed as a potential conflict of interest.

## Publisher’s Note

All claims expressed in this article are solely those of the authors and do not necessarily represent those of their affiliated organizations, or those of the publisher, the editors and the reviewers. Any product that may be evaluated in this article, or claim that may be made by its manufacturer, is not guaranteed or endorsed by the publisher.
